# Gut microbiome-Mediterranean diet interactions in improving host health

**DOI:** 10.12688/f1000research.18992.1

**Published:** 2019-05-21

**Authors:** Ravinder Nagpal, Carol A. Shively, Thomas C. Register, Suzanne Craft, Hariom Yadav

**Affiliations:** 1 Division of Internal Medicine - Molecular Medicine, Wake Forest School of Medicine, Winston-Salem, North Carolina, 27101, USA; 2 Microbiology and Immunology, Wake Forest School of Medicine, Winston-Salem, North Carolina, 27101, USA; 3 Department of Pathology - Comparative Medicine, Wake Forest School of Medicine, Winston-Salem, North Carolina, 27101, USA; 4 Department of Gerontology and Geriatric Medicine, Wake Forest School of Medicine, Winston-Salem, North Carolina, 27101, USA

**Keywords:** fiber, gut microbiota, Mediterranean diet, monkey, non-human primate, short-chain fatty acids, western diet

## Abstract

The gut microbiota plays a fundamental role in host health and disease. Host diet is one of the most significant modulators of the gut microbial community and its metabolic activities. Evidence demonstrates that dietary patterns such as the ‘Western diet’ and perturbations in gut microbiome (dysbiosis) have strong associations with a wide range of human diseases, including obesity, metabolic syndrome, type-2 diabetes and cardiovascular diseases. However, consumption of Mediterranean-style diets is considered healthy and associated with the prevention of cardiovascular and metabolic diseases, colorectal cancers and many other diseases. Such beneficial effects of the Mediterranean diet might be attributed to high proportion of fibers, mono- and poly-unsaturated fatty acids, antioxidants and polyphenols. Concurrent literature has demonstrated beneficial modulation of the gut microbiome following a Mediterranean-style diet in humans as well as in experimental animal models such as rodents. We recently demonstrated similar positive changes in the gut microbiome of non-human primates consuming a Mediterranean-style diet for long term (30 months). Therefore, it is rational to speculate that this positive modulation of the gut microbiome diversity, composition and function is one of the main factors intermediating the health effects of Mediterranean diet on the host. The present perspective discusses the evidences that the Mediterranean diet induces gut microbiome modulation in rodents, non-human primates and human subjects, and discusses the potential role of gut microbiota and microbial metabolites as one of the fundamental catalysts intermediating various beneficial health effects of Mediterranean diet on the host.

## Introduction

Diet, gut microbiome and chronic diseases like obesity, diabetes, cancer and aging-related diseases such as Alzheimer’s disease are closely associated. Our dietary habits have a strong effect on our gut microbiome and metabolism. Unsolicited perturbations in gut microbiota diversity and composition (gut dysbiosis) are key elements underlying chronic diseases including several low-grade inflammatory disorders of human gastrointestinal tract ^1^. Specifically, low consumption of dietary fibers is known to induce long-term changes in the gut microbiome that are associated with low production of beneficial microbial metabolites, i.e., short-chain fatty acids (SCFAs) such as acetate, propionate, and butyrate involved in the modulation of host immune and inflammatory health status ^2^. Epidemiological investigations have reproducibly found that the ‘Western-style’ dietary habits (typically characterized by low consumption of fruits, vegetables, salads, fish and mono- and poly-unsaturated fatty acids such as fish and olive oil, and high consumption of simple sugars, saturated fat, red meat and processed foods) as one of the main perpetrators for the rising worldwide incidence of chronic diseases ^3^. From that perspective, a dietary-pattern intervention targeting gut microbiome can provide an effective avenue for the prevention and treatment of such chronic diseases.

## Mediterranean diet: the intangible cultural and nutritional dietary pattern

**Figure 1.**
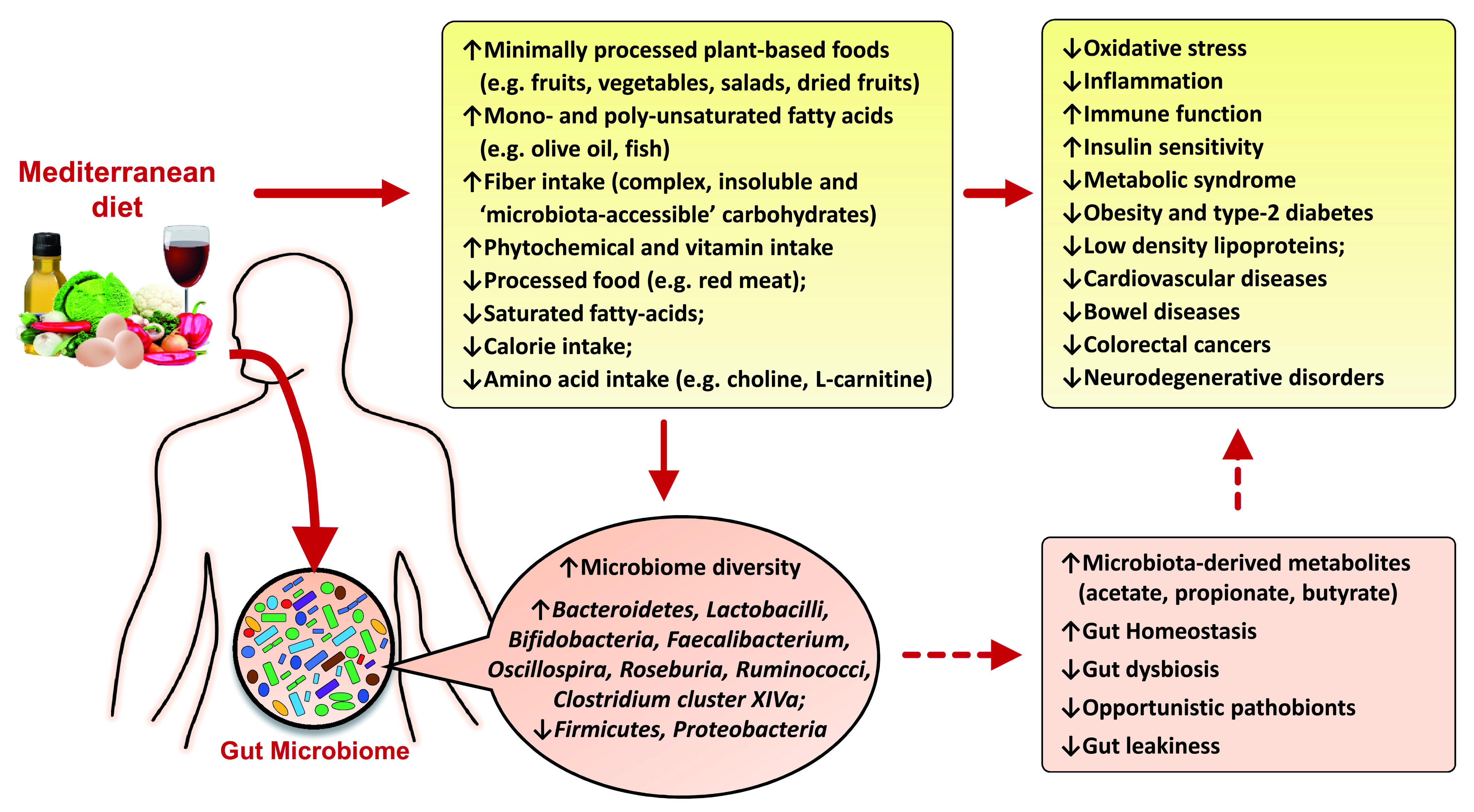
A diagrammatic overview depicting how the various health beneficial effects of Mediterranean diet might be mediated via positive changes in the gut microbiome composition and the gut microbiota-derived metabolites. References are shown in square brackets.

Prompted by this evidence, a move towards a Mediterranean-style diet is exemplified as not only a prudent choice of lifestyle but also as a scientifically accepted mechanism that is able to yield the benefits for management of several human disease pathologies and an overall improvement of health and well-being. In 2013, the Mediterranean diet (hereafter, MD) was also enrolled on the “Representative List of the Intangible Cultural Heritage of Humanity” by the United Nations Educational, Scientific and Cultural Organization (UNESCO) ^4^. In 406 B.C., Hippocrates, the father of medicine, had already stated: “Let food be your medicine and medicine be your food”. Strikingly, this 20-centuries-old statement has been clearly and consistently validated by the ever-mounting literature indicating that the dietary habits can modulate predisposition to various human gastrointestinal, metabolic, cardiovascular and systemic diseases. A Mediterranean-style diet typifies a nutritionally balanced diet, characterized by intake in high amounts and frequency of important sources of fibers (cereals, vegetables, legumes, fruits and nuts) and chemical ingredients with anti-oxidative properties (vitamins, flavonoids, phytosterols, minerals, terpenes and phenols) ( Table 1) ^5^. In addition, high proportions of oleic acid, polyphenols and unsaturated fatty acids delivers significant anti-atherogenic and anti-inflammatory properties ( Table 2) ^6^. Studies have demonstrated that switching to a Mediterranean-style diet demonstrates amelioration in serum inflammation biomarkers as well as their gene expression profile (nutrigenomics), not only in healthy subjects ^7^. but also in patients with obesity, type-2 diabetes and Crohn's disease ( Table 2) ^8– 15^. Such dietary habit changes are also accompanied by specific changes in the population level of several gut microbial groups ^6, 16– 20^. Although the association of diet-microbiome interactions with the host health and disease status remains to be comprehended by means of all-inclusive and mechanistic epidemiological and omics investigations and specific disease related pathologies; however, advancements in novel hypotheses and postulations have already exceeded over and beyond merely a speculation stage. In this context, while concurring that MD represents a promising, efficacious and holistic approach to maintain/restore host heath, it is equitable to believe that (many of) these health benefits of MD are mediated via modulation of host gut microbial clades and their metabolic functions ( Figure 1).

**Table 1.** Nutritional characteristics of a typical Mediterranean-style dietary pattern.

Ingredient	Frequency of consumption
Variety of unprocessed or minimally processed whole grains, cereals, and legumes	Regular staple
Variety of fresh fruits, vegetables, and salads	Daily basis (seasonal varieties)
Dry fruits, nuts, seeds, honey	Regular basis (generally as snacks)
Extra-virgin olive oil; fish oil	Principal source of fat (rich in poly- and mono-saturated fatty acids; low in saturated fat)
Poultry (eggs and chicken)	Low to moderate consumption
Sea food (fish, oysters, sea weeds)	Low to moderate consumption
Red and processed meats	Very low consumption
Unprocessed or minimally processed cheese and yogurt	Low to moderate consumption
Wine	Low to moderate (occasionally; particularly with evening meals)

## MD, gut microbiome and host health

The human gut microbiome is comprised of tens of trillions of microbial cells. The association of gut microbiome dysbiosis with various dramatically rising human diseases such as obesity, type-2 diabetes and aging-related diseases like Alzheimer’s disease spurs urgent need for devising effective and safe treatments using gut microbiome modulators. Our dietary practices have an immense effect on many features of gut microbes. One of the primary functions of gut microbiome is to metabolize dietary ingredients in a way that the products of this biotransformation (e.g., SCFAs, vitamins, bioactive derivative compounds and modified plant flavonoids) can benefit various aspects of human health and metabolism ^51^. Mediterranean-style diets are considered nutritionally balanced and scientifically recommended dietary pattern ( Table 1) ^5^ that are beneficial for the prevention and/or treatment of obesity, type-2 diabetes, inflammatory disorders and cardiovascular diseases ( Figure 1 and Table 2) ^8, 10, 11, 22, 52– 54^. On the other hand, consumption of Western-style diets (mainly omnivore-type diets) may lead to a gut microbiome composition that is more associated with several diseases ^55^. This increasing understanding about the importance of diet-microbiome interactions and their strong influence on human health has led to a novel and timely concept of exploring and developing ‘microbiota-directed’ foods’ that can function by modulating the functional spectrum of the gut microbial community, providing precursor substratum for microbial biotransformation to bioactive molecules beneficial for the host health, or a combination of both of these approaches ^56^. This could provide novel and potential ways for improving host health by fostering/supporting healthy gut microbial communities, restoring the loss of microbial diversity associated with Western-style diets, and ameliorating the structural and functional dysbiosis associated with various human diseases. One example of this microbiota-directed food is ‘prebiotic’, which is defined as “a non-digestible food ingredient that beneficially affects the host by selectively stimulating the growth and/or activity of one or a limited number of bacteria in the colon/gut ^57^. Indeed, a food can include one or more prebiotics (e.g., plant fibers, inulin, chicory, galacto-oligosaccharides, and others) capable of being processed by selective array of commensal/ beneficial gut microbes, leading to the production of beneficial metabolites like SCFAs. These SCFAs act as key mediators of the beneficial effects of such fibrous diets on host health ^51, 58^.

**Table 2.** Different components of a typical Mediterranean-style diet and their association with various health benefits.

MD ingredient	Positive health effect	Possible mechanism(s)	Reference
Fruits, vegetables, fiber, yogurt, probiotics/ prebiotics, vitamins	Gut homeostasis	↑Gut homeostasis ↑Beneficial gut bacteria, SCFAs ↑Gut immune system ↓Gut leakiness, opportunistic pathogens ↓Gut and systemic inflammation	16– 19, 21
PUFA and MUFA, fruits, vegetables, poultry	Cardiovascular health	↑Gut homeostasis ↑Gut permeability, beneficial gut bacteria, SCFAs ↓Gut dysbiosis ↓Gut leakiness, gut and systemic inflammation -Improved blood lipid and cholesterol profiles, ↑HDL ↓LDL and VLDL ↓Atherosclerosis	22– 28
Whole grains, fruits, vegetables, fiber, tea, polyphenols, minerals, vitamins, low- glycemic foods	Liver diseases	↑Gut homeostasis ↑Gut permeability, beneficial gut bacteria, SCFAs ↓Gut dysbiosis ↓Gut leakiness, gut and systemic inflammation ↓Liver toxicity and inflammation ↓Oxidative stress	29, 30
Fruits, vegetables, PUFA and MUFA, polyphenols, vitamins C and B _12_ and folic acid, carotenoids	Brain diseases	↑Gut homeostasis ↑Gut permeability, beneficial gut bacteria, SCFAs ↓Gut dysbiosis ↓Gut leakiness, gut and systemic inflammation ↓Gut and brain inflammation ↑Enteric nervous system ↑Memory and cognition ↓Stress, depression and hypertension	31– 33
PUFA, MUFA, nuts, antioxidants, flavonoids, yogurt, whole milk	Asthma and allergy	↑Immune-potentiation ↓Systemic inflammation ↓Oxidative stress	34– 37
Fiber, vegetables, MUFA, probiotics/ prebiotics, antioxidants, low-glycemic foods, vitamins	Diabetes	↑Gut homeostasis ↑Gut permeability, beneficial gut bacteria, SCFAs ↑Gut immune function ↓Gut leakiness ↑Immune-potentiation ↓Endotoxemia ↓Metabolic syndrome ↓Systemic low-grade inflammation ↓Oxidative stress	11– 15, 38– 40
Fiber, probiotics, PUFA, MUFA	Kidney diseases	↑Gut permeability, beneficial gut bacteria, SCFAs ↓Gut leakiness, gut and systemic inflammation ↑Saccharolytic gut bacteria ↓Proteolytic gut bacteria ↓p-cresol indoxyl sulphate	41– 43
Fresh fruits and vegetables, vitamins C and E, β-carotene	Gastric disorders	↑Gastric homeostasis ↓Opportunistic pathogens ↓Chemical contaminants ↓Oxidative stress ↓Ulcers	44
Fiber, PUFA/MUFA, probiotics/prebiotics, antioxidants, vitamins, polyphenols	Colorectal cancers	↑Immune-function ↓Low-grade inflammation ↓Oxidative stress	24, 44– 48
Fiber, probiotics/prebiotics, omega-3 fatty acids, antioxidants, low-glycemic index, polyphenols	Obesity/ Weight loss	↑Gut homeostasis ↑Gut permeability, beneficial gut bacteria, SCFAs ↓Gut leakiness ↑Insulin sensitivity ↑GLP-1, PYY ↑Satiety ↓Colonic transit ↓Fasting glucose, C-peptide ↓IGF-1 ↓Nutrient sensing ↓Oxidative stress	49, 50

GLP-1, glucagon-like peptide 1; HDL, high-density lipoproteins; IGF, insulin-like growth factor; LDL, low-density lipoproteins; MUFA, monounsaturated fatty acids; PUFA, polyunsaturated fatty acids; PYY, peptide YY; SCFAs, short-chain fatty acids; VLDL, very low density lipoproteins.

Higher adherence to MD has been found to be associated with reduced incidences of several chronic diseases, such as obesity, type-2 diabetes, metabolic syndrome, gastrointestinal cancer, cardiovascular diseases, fatty liver diseases, chronic kidney diseases and neurodegenerative diseases like Alzheimer’s disease ( Table 2) ^59– 63^. Based on different studies, the lowering effects of MD against the incidence of these disorders have been attributed to different MD dietary constituents derived from fruits, vegetables, lean meat, nuts and grains, fibers, mono- and poly-unsaturated fatty acids, vitamins, polyphenols, and antioxidant ( Table 2) ^64– 66^. Considering that the pathology of the above-mentioned diseases involves a dysbiotic gut microbiome and that the ingredients of the MD closely interact with gut microbial community, it can be suggested that the mechanisms by which MD drives health effects on the host might be mediated by an integrated interplay between the diet and the gut microbiome ( Figure 1). Two main mechanisms underlying these positive effects of MD are (a) beneficial modulation of the gut microbiota (i.e., increased microbial diversity and production of beneficial metabolites like SCFAs), and (b) reduced metabolic endotoxemia by suppressing the growth of gram-negative bacteria and improving the gut barrier integrity by modulating tight junctions and mucus secretion ^67^. Both of these mechanisms can help in reducing the systemic inflammation, which is the hallmark of many chronic diseases in the human body. Table 2 presents some of the reported beneficial health effects of Mediterranean-style diets and summarizes how different MD components could prevent/ameliorate these disorders by promoting a homeostatic gut microbiome and permeability to maintain the balanced arrays of the bacterial metabolites and the inflammatory molecules across the gut epithelium. 

## Microbiome-mediated pro-health effects of MD: purported and speculated mechanisms

Extensive literature is available about the health benefits of MD; however, knowledge concerning the impact of MD on gut microbiota-mediated disease outcomes remain limited. The precise mechanism by which MD confers its beneficial effects on host health such as lowering the risk of cardiovascular, metabolic and gastrointestinal diseases and certain cancers remains unclear. However, most of these beneficial effects have long been speculated to be mediated by several interrelated and overlapping factors including cholesterol-lowering effect, protection against oxidative stress and inflammation, modification of hormones/growth factors involved in carcinogenesis, and control/inhibition of nutrient-sensing pathways by restriction of specific amino acids ^66^. In addition, recent literature suggests that diet has a major impact on gut microbiome and the production of gut microbiota-derived microbial metabolites, which can considerably influence the host metabolic health ^68^. Ever-mounting metagenomic studies have suggested that specific nutrients, particularly dietary fiber and proteins, exert strong effects on gut microbiota composition as well as on the production of microbial metabolites that further influence multiple features of the host immune and metabolic health ^69, 70^. For example, trimethylamine N-oxide (TMAO), a gut microbial metabolites from dietary choline and L-carnitine, is known to increase the risk of cardiovascular diseases independently of cardio-metabolic risk factors ^71^. TMAO, at abnormally high levels, can induce vascular inflammation and prothrombosis by exaggerating platelet hyper-responsiveness to multiple agonists, and could be implicated in the pathogenesis of obesity and type 2 diabetes ^72, 73^. Given that a typical Mediterranean-style diet contains a proportion of choline and L-carnitine (which are abundant in eggs, red meat, and cheese) that is over 50% lower than a typical Western-style diet, it could be speculated that some of the beneficial effects of MD on host cardio-metabolic health might at least partly be mediated via such microbiome-related mechanisms. On the other hand, studies have shown that a poor adherence to a Mediterranean-style diet is associated with higher urinary TMAO level ^16^. It is acknowledged that the physiological effects of the gut microbiota on host health are mediated not only by the direct interaction of the microbe with the host but also equivalently by the indirect microbial processes including the production of fermentation metabolites from diet or *de novo*. Thus, the array of microbial metabolites in the host gut from dietary fermentation is a function of not only the gut microbial ecology but also the substrate such as the fermentation of complex carbohydrates (e.g., fiber) or other plant-based foods, which are abundant in a Mediterranean-style diet, that leads to the production of beneficial SCFAs, in contrast to TMAO, which is frequently observed in people consuming Western-style diets poor in minimally processed plant-based foods and rich in processed red meats.

MD is also rich in complex and insoluble fiber content when compared to a typical Western-style diet. A high intake of dietary fiber is well known to promote the beneficial modulation/maintenance of the gut microbiota with a reduced population of Firmicutes, while increasing that of Bacteroidetes, thereby yielding high levels of SCFAs including butyrate in the gut. These microbiota-derived metabolites including acetate, propionate and butyrate are known to protect against the development of several intestinal, inflammatory and allergic disease, and some of these effects are thought to be mediated via binding of these metabolites to specific G-protein-coupled receptors expressed on enteroendocrine and immune cells ^74^. Adherence to a Mediterranean-style diet has been shown to reshape the gut microbiota of obese individuals, with increased population of *Bacteroides, Prevotella, Roseburia, Ruminococcus,* and *Faecalibacterium prausnitzii*, which are known for their fibrolytic activity, producing SCFAs by metabolizing carbohydrates ^18^. Notably, *Bacteroides fragilis* and *F. prausnitzii* are also known to confer anti-inflammatory effects via inducing CD4+ T cells, the secretors of the anti-inflammatory interleukin-10 ^75, 76^. In addition, the high content of vegetables, legumes, and fruit in a Mediterranean-style diet are also found to be associated with increased intestinal levels of short-chain fatty acids. These reports suggest that the Mediterranean-style diet fosters beneficial bacteria in the gut, which subsequently secretes beneficial metabolites.

Another reason that could underlie the health beneficial effects of Mediterranean-style diet is the lower intake of processed foods, as the Mediterranean dietary pattern is rich in minimally processed food, unlike a Western-style dietary pattern, which contains a much higher proportion of processed plant- and animal-based foods. This minimized intake of processed plant food with restricted calorie intake through MD is also known to confer positive effects on the gut microbiota diversity and composition by protecting/fostering the homeostasis of population levels of several beneficial bacterial groups ^77^. On the other hand, long-term adherence to a Western-style dietary pattern, which has very low content of complex, insoluble, minimally processed, and microbiota-accessible plant-based fibers and carbohydrates, can lead to a dysbiotic gut microbiota, with various subdominant but important bacterial groups vanishing over generations, and may also negatively influence several important features of the host immune system, thereby increasing the predisposition to various gastrointestinal, metabolic and immune diseases ^74, 76, 78^. Several studies have shown that the healthier and calorie-restricted dietary patterns with a high proportion of minimally processed plant-based foods can reprogram several important gut microbial functions that are crucial for promoting host health and wellbeing ^77, 79^.

Emerging evidence shows that the adherence to a MD is associated with higher gut microbial diversity. The CORonary Diet Intervention with Olive Oil and Cardiovascular PREVention (CORDIOPREV) study involving 138 participants with metabolic syndrome and 101 healthy counterparts has reported restoration of the gut microbial dysbiosis in metabolic syndrome patients following long-term adherence to the MD, although the disease persisted ^80^. Interestingly, the study demonstrated that the MD adherence could restore the patients’ gut microbiota in a similar way to the gut microbiota composition seen in metabolically healthy subjects, by fostering the population of saccharolytic bacterial genera, including *Bacteroidetes*, *Faecalibacterium*, *Roseburia* and *Ruminococci*, all of which are known to be associated with increased fermentation capacity to produce healthy SCFAs in the gut. A long-term adherence to the MD has also been found to increase the population of *Roseburia* sp. and *Oscillospira* sp., in addition to improving insulin sensitivity in obese people ^81^. Notably, *Roseburia* is a prominent butyrate-producing genus that has been found to confer anti-inflammatory effects and is generally found to be reduced in type-2 diabetes patients ^82, 83^. These reports suggest that a MD might be effective in the prevention and management of type-2 diabetes, although more studies are needed to affirm these therapeutic effects in particular reference to their connection with gut microbiota-associated factors.

Altogether, these reports suggest that the MD modulates gut microbiota profiles (such as higher population levels of *Bacteroidetes, Clostridium* cluster XIVa, *Faecalibacterium prausnitzii, Lactobacilli,* and *Bifidobacteria*, or lower Firmicutes) and can influence the diversity, activities and functionalities of various gut bacteria, thereby also fostering healthy metabolites (such as SFCAs) that can confer multiple benefits to the host intestinal, metabolic and immune health. Nevertheless, broader and more inclusive clinical and epidemiological studies assessing the temporal changes in the composition and function of gut microbiota enterotypes are still needed to establish a gut microbiome signature as a marker of MD adherence.

## Non-human primates (NHPs): an ideal model to elucidate diet-microbiome interactions in human health and disease

Given the profound impact of diet on the diversity and composition of host gastrointestinal microbiome ^68, 84– 86^, the gut microbiome can be illustrated as a valuable biomarker of long-term intake of healthy or unhealthy diets. Therefore, it is imperative to elucidate whether, how and to what extent these long-term dietary patterns can influence the composition of the gut microbiota and how this could affect the production of beneficial microbial metabolites. As mentioned above, diet shapes the gut microbiome by supplying specific substrates that differentially foster the growth of specific gut microbial communities ^87– 89^; this diet-microbiome network is universally consistent in human and animal studies ^85, 87, 90, 91^. Specifically, the majority of diet-microbiome-targeted studies focus on high-fat, high-sugar, low-fiber vs. low-fat, low-sugar, high-fiber diets, with particular reference to nutrition- and gut-related maladies, including obesity, endotoxemia, insulin resistance, type-2 diabetes, and other metabolic syndromes ^92, 93^. Several animal models, including those in mice, hamsters, rats, guinea pigs and zebrafish are used for investigating diet-microbiome interactions, but, given the specific and prominent impact of dietary patterns on the gut microbiome, these models might not invariably be truly translatable to human milieus owing to far-reaching dissimilarities in their dietary regimen, age, body size and environmental elements. Accordingly, novel models such as NHPs are being sought for investigations of human diets and their interactions with gut microbiome, to decipher better understanding in human inference ^92, 94– 100^. In addition, while it is challenging to precisely control and monitor dietary patterns through questionnaires in human interventions, studies performed on small animals are disadvantaged by short-term diet intervention periods. To overcome these limitations, NHPs represent an excellent model for investigating the diet-microbiome interactions and their impact on host health. Their physiological and phylogenetic closeness to humans makes them a perfect and biologically relevant animal model for human context to examine diet-microbiome connections as well as for studying the relation of this network with different nutrition- and gut-related diseases ^95, 96, 99– 102^. In one such endeavor, we recently performed a study wherein we demonstrated the effect of a long-term Western- vs. Mediterranean-style diet intake on the gut microbiome composition in NHPs (Cynomolgus monkeys; *Macaca fascicularis*) ^21^. Previous reports have demonstrated that the gut microbiome of NHPs resembles more closely to those of primates than other animals ^103^. The human gut largely harbors microbes belonging to nine different bacterial divisions, viz. Firmicutes, Bacteroides, Actinobacteria, Proteobacteria, Verrucomicrobia, Fusobacteria, Spirochaetes, Cyanobacteria, and VadinBE97 ^104, 105^. Interestingly, our NHP data also demonstrated Firmicutes, Bacteroidetes, Proteobacteria, Actinobacteria, Verrucomicrobia, Fibrobacteres, Spirochaetes, Cyanobacteria, and Tenericutes as most abundant bacterial phyla ^21^. This again validates that the NHP microbiome studies can yield important indications about specific features of these bacterial clades in the human gut and provide unique opportunities to investigate diet-microbiome interactions.

## Effect of MD on NHP microbiome: similar findings as seen in human and rodent studies

**Figure 2.**
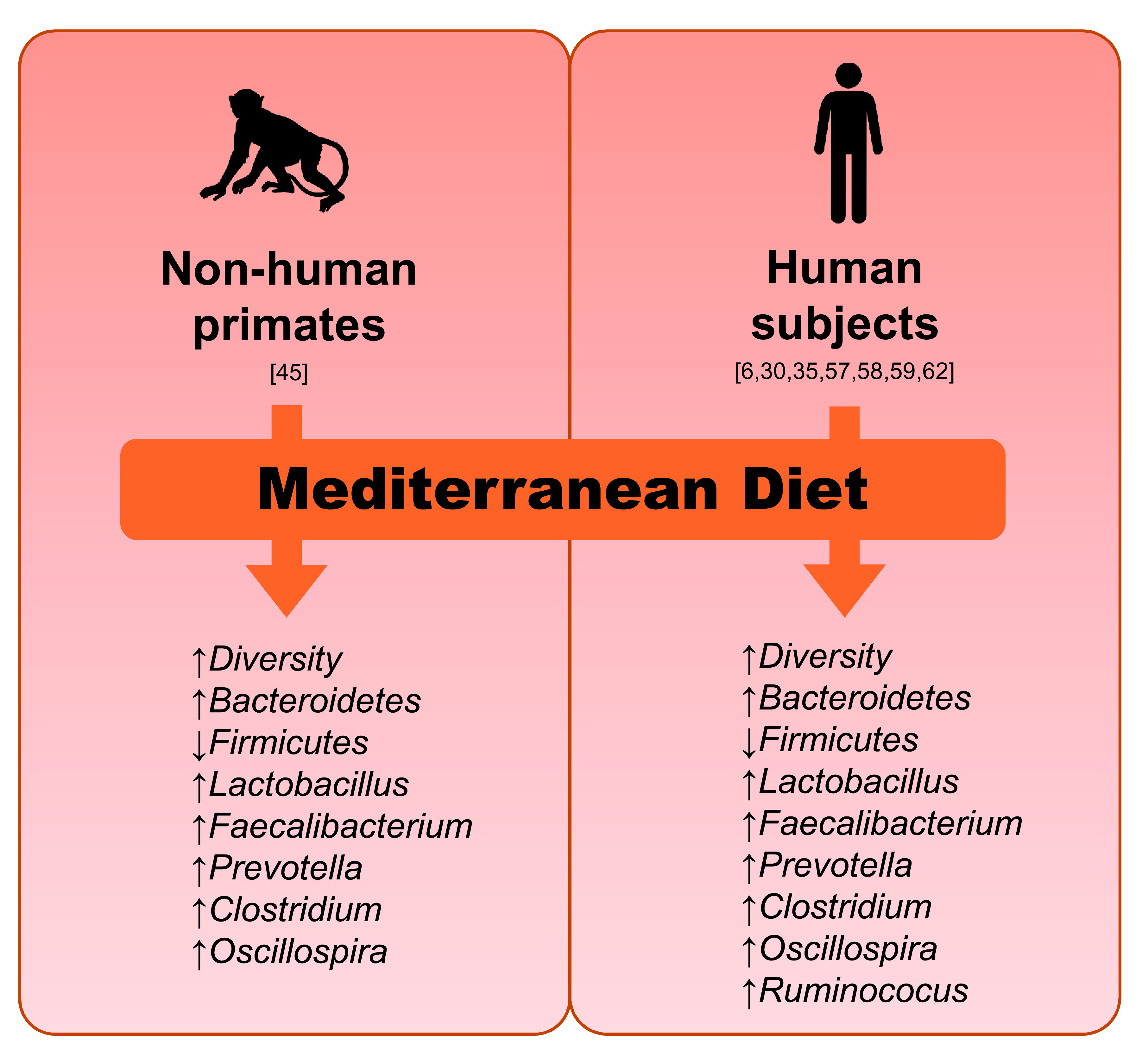
Effects of Mediterranean diet on the population of major gut bacterial groups that have been reported consistently in different studies involving non-human primates and human subjects.

Our data obtained feeding a MD to NHPs also demonstrated that MD boosted the gut microbiome diversity and promoted the carriage of several important bacterial groups, including *Bacteriodes*, *Prevotella*, *Lactobacillus*, *Faecalibacterium*, *Clostridium* and *Oscillospira* ^21^, again corroborating that the host diet can influence gut microbiome diversity and composition. Notably, similar effects as seen in the NHP cohort have also previously been seen in human studies ( Figure 2). For example, diets rich in fiber and unsaturated fatty acids are known to help maintaining a healthy and diverse gut microbiome. Our NHP data also demonstrated higher alpha-diversity in NHPs consuming the MD, an effect that has also been observed in several other studies on humans or animal animals ^68, 78, 99, 106^ and can be attributed to a higher proportion of fiber in the MD. In addition, our NHP study also demonstrated higher Bacteroidetes-Firmicutes ratio and higher abundance of *Clostridium* and *Prevotella* in NHPs on MD. Similar results have been reported by previous studies performed in humans and other mammals reporting that MD (and specifically the higher fiber intake and less intake of high-glycemic index sugars) positively alters the gut microbiota, with an increased *Bacteroides*, *Clostridium* and *Prevotella* population ^8, 88, 99, 107– 109^ whereas a low-fiber, high-fat and high-sugar diet is linked with increased *Firmicutes* and reduced *Bacteroides* population ^88^. Another interesting observation was the increased abundance of genus *Oscillospira* in MD-fed NHPs. *Oscillospira,* a genus from the *Ruminococcaceae* family, is usually found prevalent in the gastrointestinal tract of ruminants consuming diets rich in complex plant fibers and hence is regarded as genus adapted to vegetable-rich diets, such as Mediterranean-style diets ^110, 111^.

Another interesting observation from this NHP study was the increased abundance of *Faecalibacterium prausnitzii,* one of the beneficial butyrate-producer previously reported to be found in higher numbers in humans consuming a Mediterranean-style diet ^111– 113^. Various human and small animal studies have reproducibly reported fostering of a population of beneficial bacterial groups, including *Lactobacillus* sp ^114– 116^. and *Faecalibacterium* sp ^117^. following consumption of diets that are rich in complex carbohydrates, such as prebiotics. In addition, omega-3 fatty acids, which are present in higher ratio in Mediterranean-style diet, can also stimulate the intestinal population of several beneficial bacteria including lactobacilli that typically inhabit the distal gut, a primary site for the metabolism of mono- and poly-unsaturated fatty acids ^9, 118, 119^. Interestingly, in a recent study on these NHPs, we demonstrated that the consumption of MD also leads to an increased *Lactobacillus* abundance and increased bacterially processed bioactive compounds in the mammary glands compared with Western diet-fed monkeys, clearly indicating that the influence of MD on the microbiome reaches far outside the gut in distal sites such as the mammary glands and could also establish an alternative pathway for breast cancer prevention ^120^. Conversely, high-fat diets are also known to reduce the intestinal carriage of lactobacilli ^93, 121, 122^. In addition, we have recently reported that MD also protects these NHPs against hepatosteatosis while reducing BMI and body fat ^123^. Hence, these common findings between NHP and human studies clearly demonstrate and advocate that NHPs can prove to be an excellent experimental animal model to examine the diet-microbiome interaction in context to human health and disease.

Altogether, these findings from our cohort of the MD-fed NHPs demonstrate that the positive modulation of the gut microbiome is concomitant with the beneficial effects of MD on host health and hint that this microbiome modulation might even at least in part also mediate/underlie the health effects of MD, particularly given that the majority of the MD-induced changes in the gut microbiome including enhanced microbial diversity and fostering of several beneficial bacterial groups are known to be beneficial for host intestinal, metabolic and overall health ^21^. However, although the health benefits of MD have been studied extensively, research on MD’s impact on microbiome-mediated disease outcomes still remains undetermined. For instance, it is known that MD consumption leads to gut microbiome alterations; however, the magnitude to which these alterations occur may be potentially impacted by multiple factors such as the study duration, host age and lifestyle habits, specific disease predisposition or severity, level of dietary adherence, etc. which otherwise remain to be explicated and hence would be prerequisite to pinpoint the factors contributing to the outcome of MD on gut microbiome and deliver conclusive data on the role of gut microbiome in MD’s beneficial health outcomes ^124, 125^. As a result, according to the existing literature, the examination of gut microbial composition could not be endorsed as a stand-alone tool for the health effects of MD. To this end, systemic approaches that incorporate gut metagenomics, transcriptomics and metabolomics could help elucidate the complex association of MD-microbiome interrelationship with host health.

## Perspectives on the future

Recent evidences from animal and human studies are beginning to elucidate the mechanisms underlying the pro-health effects of the traditional MD. The metabolism and fermentation of plant-based foods packed with complex fibers, a wide variety of vitamins and phytochemicals, could also play an important role in promoting host metabolic health. Although more research is needed, specific dietary patterns (particularly, the Mediterranean-style diet) have been found to have a strong influence on the gut microbiome composition and metabolic function, suggesting that there is an opportunity to prevent and treat various lifestyle-related disorders based on gut microbiota outcomes. Given the plasticity of the gut microbiota, it can be speculated that such Mediterranean-style nutritional intervention can positively affect host health, either by maintaining a physiologically homeostatic gut microbiota configuration or by reversing the gut dysbiosis state; this positive modulation of the gut microbiome could be considered as a potential and holistic target for non-pharmacological interventions in a variety of disease states. The potentiality of the gut microbiome is tremendous. It can be easily and rapidly modulated in a natural, non-invasive, non-pharmacologic way, i.e., via diets and other dietary supplements like probiotics and prebiotics. The ever-mounting knowledge of the gut microbiome as acquired by the use of remarkable high-throughput sequencing and omics tools in combination with the data from various *in vitro* and *in vivo* experimental models is shedding a new light on the key mechanisms through which diet-microbiome crosstalk in the gut can modulate potential risk factors of several human diseases. This wealth of knowledge is certainly going to offer potential avenues for decoding the complexities and functionalities of the diet-microbiome interface and for developing personalized dietary strategies to determine the healthiest dietary pattern for the personalized host.

In conjunction with gut microbial composition in response to diets like MD and the Western diet, it is becoming evident that the microbial metabolites produced by gut microbiome and dietary interactions plays an important role to regulate host metabolism and physiology ^126^. MD feeding enhances abundance of probiotics or beneficial bacteria like lactobacilli ^21^, however, future studies focusing on determining how the increased abundance of lactobacilli benefit host health will be important to conclude the mechanistic views. It is difficult to pinpoint exact molecular mechanism(s) by which diets and ingredients can impact human health; however, several layers of mechanistic studies like systemic, organ- and cell-based mechanism should be the focus of future research. Such studies can provide important information to include the vital ingredients that not only enhance the impact of diets like MD, but can easily be tailored for personalized nutritional approaches. Combining gut microbiome, and metabolic response (like the glycemic index) of diets on individual basis are attractive area of current research, that can extend valuable outcome in the field of diet-microbiome interactions to benefit human health.

## Data availability

No data are associated with this article.

## References

[1] Brown K DeCoffe D Molcan E: Diet-induced dysbiosis of the intestinal microbiota and the effects on immunity and disease. *Nutrients.* 2012;4(8):1095–1119. 10.3390/nu4081095 23016134 PMC3448089

[2] Maslowski KM Mackay CR: Diet, gut microbiota and immune responses. *Nat Immunol.* 2011;12(1):5–9. 10.1038/ni0111-5 21169997

[3] Devereux G: The increase in the prevalence of asthma and allergy: food for thought. *Nat Rev Immunol.* 2006;6(11):869–74. 10.1038/nri1958 17063187

[4] UNESCO: Mediterranean diet.2013 Reference Source

[5] Trichopoulou A Kouris-Blazos A Wahlqvist ML: Diet and overall survival in elderly people. *BMJ.* 1995;311(7018):1457–1460. 10.1136/bmj.311.7018.1457 8520331 PMC2543726

[6] Del Chierico F Vernocchi P Dallapiccola B: Mediterranean diet and health: food effects on gut microbiota and disease control. *Int J Mol Sci.* 2014;15(7):11678–11699. 10.3390/ijms150711678 24987952 PMC4139807

[7] Ellett S: in Nutrigenomics and nutrigenetics in functional foods and personalized nutrition. (CRC Press).2016;286–297.

[8] Marlow G Ellett S Ferguson IR: Transcriptomics to study the effect of a Mediterranean-inspired diet on inflammation in Crohn's disease patients. *Hum Genomics.* 2013;7:24. 10.1186/1479-7364-7-24 24283712 PMC4174666

[9] Kaliannan K Wang B Li XY: Omega-3 fatty acids prevent early-life antibiotic exposure-induced gut microbiota dysbiosis and later-life obesity. *Int J Obes (Lond).* 2016;40(6):1039–42. 10.1038/ijo.2016.27 26876435

[10] Lopez-Legarrea P Fuller NR Zulet MA: The influence of Mediterranean, carbohydrate and high protein diets on gut microbiota composition in the treatment of obesity and associated inflammatory state. *Asia Pac J Clin Nutr.* 2014;23(3):360–368. 10.6133/apjcn.2014.23.3.16 25164445

[11] Salas-Salvadó J Bulló M Babio N: Reduction in the incidence of type 2 diabetes with the Mediterranean diet: results of the PREDIMED-Reus nutrition intervention randomized trial. *Diabetes Care.* 2011;34(1):14–19. 10.2337/dc10-1288 20929998 PMC3005482

[12] Salas-Salvadó J Bulló M Estruch R: Prevention of diabetes with Mediterranean diets: a subgroup analysis of a randomized trial. *Ann Intern Med.* 2014;160(1):1–10. 10.7326/M13-1725 24573661

[13] Martínez-González MA de la Fuente-Arrillaga C Nunez-Cordoba JM: Adherence to Mediterranean diet and risk of developing diabetes: prospective cohort study. *BMJ.* 2008;336(7657):1348–1351. 10.1136/bmj.39561.501007.BE 18511765 PMC2427084

[14] Esposito K Maiorino MI Di Palo C: Adherence to a Mediterranean diet and glycaemic control in Type 2 diabetes mellitus. *Diabet Med.* 2009;26(9):900–907. 10.1111/j.1464-5491.2009.02798.x 19719711

[15] Tortosa A Bes-Rastrollo M Sanchez-Villegas A: Mediterranean diet inversely associated with the incidence of metabolic syndrome: the SUN prospective cohort. *Diabetes Care.* 2007;30(11):2957–2959. 10.2337/dc07-1231 17712023

[16] De Filippis F Pellegrini N Vannini L: High-level adherence to a Mediterranean diet beneficially impacts the gut microbiota and associated metabolome. *Gut.* 2016;65(11):1812–1821. 10.1136/gutjnl-2015-309957 26416813

[17] Mitsou EK Kakali A Antonopoulou S: Adherence to the Mediterranean diet is associated with the gut microbiota pattern and gastrointestinal characteristics in an adult population. *Br J Nutr.* 2017;117(12):1645–1655. 10.1017/S0007114517001593 28789729

[18] Haro C García-Carpintero S Rangel-Zúñiga OA: Consumption of Two Healthy Dietary Patterns Restored Microbiota Dysbiosis in Obese Patients with Metabolic Dysfunction. *Mol Nutr Food Res.* 2017;61(12):1700300. 10.1002/mnfr.201700300 28940737

[19] Garcia-Mantrana I Selma-Royo M Alcantara C: Shifts on Gut Microbiota Associated to Mediterranean Diet Adherence and Specific Dietary Intakes on General Adult Population. *Front Microbiol.* 2018;9:890. 10.3389/fmicb.2018.00890 29867803 PMC5949328

[20] Pignanelli M Just C Bogiatzi C: Mediterranean Diet Score: Associations with Metabolic Products of the Intestinal Microbiome, Carotid Plaque Burden, and Renal Function. *Nutrients.* 2018;10(6): pii: E779. 10.3390/nu10060779 29914158 PMC6024790

[21] Nagpal R Shively CA Appt SA: Gut Microbiome Composition in Non-human Primates Consuming a Western or Mediterranean Diet. *Front Nutr.* 2018;5:28. 10.3389/fnut.2018.00028 29922651 PMC5996930

[22] Estruch R Ros E Salas-Salvadó J: Primary prevention of cardiovascular disease with a Mediterranean diet. *N Engl J Med.* 2013;368(14):1279–1290. 10.1056/NEJMoa1200303 23432189

[23] Georgousopoulou EN Kastorini CM Milionis HJ: Association between Mediterranean diet and non-fatal cardiovascular events, in the context of anxiety and depression disorders: a case/case-control study. *Hellenic J Cardiol.* 2014;55(1):24–31. 24491932

[24] Pauwels EK: The protective effect of the Mediterranean diet: focus on cancer and cardiovascular risk. *Med Princ Pract.* 2011;20(2):103–111. 10.1159/000321197 21252562

[25] Kaliora AC Dedoussis GV Schmidt H: Dietary antioxidants in preventing atherogenesis. *Atherosclerosis.* 2006;187(1):1–17. 10.1016/j.atherosclerosis.2005.11.001 16313912

[26] Dontas AS Zerefos NS Panagiotakos DB: Mediterranean diet and prevention of coronary heart disease in the elderly. *Clin Interv Aging.* 2007;2(1):109–15. 18044083 10.2147/ciia.2007.2.1.109 PMC2684076

[27] Panagiotakos DB Dimakopoulou K Katsouyanni K: Mediterranean diet and inflammatory response in myocardial infarction survivors. *Int J Epidemiol.* 2009;38(3):856–866. 10.1093/ije/dyp142 19244256

[28] Pérez-Jiménez F López-Miranda J Mata P: Protective effect of dietary monounsaturated fat on arteriosclerosis: beyond cholesterol. *Atherosclerosis.* 2002;163(2):385–398. 10.1016/S0021-9150(02)00033-3 12052487

[29] Abenavoli L Di Renzo L Boccuto L: Health benefits of Mediterranean diet in nonalcoholic fatty liver disease. *Expert Rev Gastroenterol Hepatol.* 2018;12(9):873–881. 10.1080/17474124.2018.1503947 30033779

[30] Salamone F Li Volti G Titta L: *Moro* orange juice prevents fatty liver in mice. *World J Gastroenterol.* 2012;18(29):3862–8. 10.3748/wjg.v18.i29.3862 22876038 PMC3413058

[31] Féart C Samieri C Allès B: Potential benefits of adherence to the Mediterranean diet on cognitive health. *Proc Nutr Soc.* 2013;72(1):140–152. 10.1017/S0029665112002959 23228285

[32] Féart C Samieri C Rondeau V: Adherence to a Mediterranean diet, cognitive decline, and risk of dementia. *JAMA.* 2009;302(6):638–648. 10.1001/jama.2009.1146 19671905 PMC2850376

[33] Valls-Pedret C Lamuela-Raventós RM Medina-Remón A: Polyphenol-rich foods in the Mediterranean diet are associated with better cognitive function in elderly subjects at high cardiovascular risk. *J Alzheimers Dis.* 2012;29(4):773–782. 10.3233/JAD-2012-111799 22349682

[34] Garcia-Marcos L Castro-Rodriguez JA Weinmayr G: Influence of Mediterranean diet on asthma in children: a systematic review and meta-analysis. *Pediatr Allergy Immunol.* 2013;24(4):330–338. 10.1111/pai.12071 23578354

[35] Arvaniti F Priftis KN Papadimitriou A: Adherence to the Mediterranean type of diet is associated with lower prevalence of asthma symptoms, among 10-12 years old children: the PANACEA study. *Pediatr Allergy Immunol.* 2011;22(3):283–289. 10.1111/j.1399-3038.2010.01113.x 21457335

[36] Castro-Rodriguez JA Garcia-Marcos L Sanchez-Solis M: Olive oil during pregnancy is associated with reduced wheezing during the first year of life of the offspring. *Pediatr Pulmonol.* 2010;45(4):395–402. 10.1002/ppul.21205 20306538

[37] Chatzi L Apostolaki G Bibakis I: Protective effect of fruits, vegetables and the Mediterranean diet on asthma and allergies among children in Crete. *Thorax.* 2007;62(8):677–83. 10.1136/thx.2006.069419 17412780 PMC2117278

[38] Marrazzo G Barbagallo I Galvano F: Role of dietary and endogenous antioxidants in diabetes. *Crit Rev Food Sci Nutr.* 2014;54(12):1599–1616. 10.1080/10408398.2011.644874 24580561

[39] Paniagua JA de la Sacristana AG Sánchez E: A MUFA-rich diet improves posprandial glucose, lipid and GLP-1 responses in insulin-resistant subjects. *J Am Coll Nutr.* 2007;26(5):434–444. 10.1080/07315724.2007.10719633 17914131

[40] Rocca AS LaGreca J Kalitsky J: Monounsaturated fatty acid diets improve glycemic tolerance through increased secretion of glucagon-like peptide-1. *Endocrinology.* 2001;142(3):1148–1155. 10.1210/endo.142.3.8034 11181530

[41] Montemurno E Cosola C Dalfino G: What would you like to eat, Mr CKD Microbiota? A Mediterranean Diet, please! *Kidney Blood Press Res.* 2014;39(2–3):114–123. 10.1159/000355785 25117687

[42] Mekki K Bouzidi-bekada N Kaddous A: Mediterranean diet improves dyslipidemia and biomarkers in chronic renal failure patients. *Food Funct.* 2010;1(1):110–115. 10.1039/c0fo00032a 21776461

[43] Ranganathan N Ranganathan P Friedman EA: Pilot study of probiotic dietary supplementation for promoting healthy kidney function in patients with chronic kidney disease. *Adv Ther.* 2010;27(9):634–647. 10.1007/s12325-010-0059-9 20721651

[44] Praud D Bertuccio P Bosetti C: Adherence to the Mediterranean diet and gastric cancer risk in Italy. *Int J Cancer.* 2014;134(12):2935–2941. 10.1002/ijc.28620 24259274

[45] Donovan MG Selmin OI Doetschman TC: Mediterranean Diet: Prevention of Colorectal Cancer. *Front Nutr.* 2017;4:59. 10.3389/fnut.2017.00059 29259973 PMC5723389

[46] Couto E Boffetta P Lagiou P: Mediterranean dietary pattern and cancer risk in the EPIC cohort. *Br J Cancer.* 2011;104(9):1493–9. 10.1038/bjc.2011.106 21468044 PMC3101925

[47] Cappellani A Zanghì A Di Vita M: Strong correlation between diet and development of colorectal cancer. *Front Biosci (Landmark Ed).* 2013;18:190–198. 23276917 10.2741/4095

[48] Biondi A Fisichella R Fiorica F: Food mutagen and gastrointestinal cancer. *Eur Rev Med Pharmacol Sci.* 2012;16(9):1280–1282. 23047513

[49] Shai I Schwarzfuchs D Henkin Y: Weight loss with a low-carbohydrate, Mediterranean, or low-fat diet. *N Engl J Med.* 2008;359(3):229–241. 10.1056/NEJMoa0708681 18635428

[50] Kaaks R Bellati C Venturelli E: Effects of dietary intervention on IGF-I and IGF-binding proteins, and related alterations in sex steroid metabolism: the Diet and Androgens (DIANA) Randomised Trial. *Eur J Clin Nutr.* 2003;57(9):1079–88. 10.1038/sj.ejcn.1601647 12947426

[51] Koh A De Vadder F Kovatcheva-Datchary P: From Dietary Fiber to Host Physiology: Short-Chain Fatty Acids as Key Bacterial Metabolites. *Cell.* 2016;165(6):1332–1345. 10.1016/j.cell.2016.05.041 27259147

[52] Santoro A Pini E Scurti M: Combating inflammaging through a Mediterranean whole diet approach: the NU-AGE project's conceptual framework and design. *Mech Ageing Dev.* 2014;136–137:3–13. 10.1016/j.mad.2013.12.001 24342354

[53] Craig WJ: Health effects of vegan diets. *Am J Clin Nutr.* 2009;89(5):1627S–1633S. 10.3945/ajcn.2009.26736N 19279075

[54] Craig WJ: Nutrition concerns and health effects of vegetarian diets. *Nutr Clin Pract.* 2010;25(6):613–620. 10.1177/0884533610385707 21139125

[55] Albenberg LG Wu GD: Diet and the intestinal microbiome: associations, functions, and implications for health and disease. *Gastroenterology.* 2014;146(6):1564–1572. 10.1053/j.gastro.2014.01.058 24503132 PMC4216184

[56] Barratt MJ Lebrilla C Shapiro HY: The Gut Microbiota, Food Science, and Human Nutrition: A Timely Marriage. *Cell Host Microbe.* 2017;22(2):134–141. 10.1016/j.chom.2017.07.006 28799899 PMC5915309

[57] Gibson GR Roberfroid MB: Dietary modulation of the human colonic microbiota: introducing the concept of prebiotics. *J Nutr.* 1995;125(6):1401–1412. 10.1093/jn/125.6.1401 7782892

[58] Joseph J Depp C Shih PB: Modified Mediterranean Diet for Enrichment of Short Chain Fatty Acids: Potential Adjunctive Therapeutic to Target Immune and Metabolic Dysfunction in Schizophrenia? *Front Neurosci.* 2017;11:155. 10.3389/fnins.2017.00155 28396623 PMC5366345

[59] Schwingshackl L Schwedhelm C Galbete C: Adherence to Mediterranean Diet and Risk of Cancer: An Updated Systematic Review and Meta-Analysis. *Nutrients.* 2017;9(10): pii: E1063. 10.3390/nu9101063 28954418 PMC5691680

[60] Grosso G Marventano S Yang J: A comprehensive meta-analysis on evidence of Mediterranean diet and cardiovascular disease: Are individual components equal? *Crit Rev Food Sci Nutr.* 2017;57(15):3218–3232. 10.1080/10408398.2015.1107021 26528631

[61] Schwingshackl L Missbach B König J: Adherence to a Mediterranean diet and risk of diabetes: a systematic review and meta-analysis. *Public Health Nutr.* 2015;18(7):1292–1299. 10.1017/S1368980014001542 25145972 PMC10273006

[62] Sofi F Abbate R Gensini GF: Accruing evidence on benefits of adherence to the Mediterranean diet on health: an updated systematic review and meta-analysis. *Am J Clin Nutr.* 2010;92(5):1189–1196. 10.3945/ajcn.2010.29673 20810976

[63] Mitrou PN Kipnis V Thiébaut AC: Mediterranean dietary pattern and prediction of all-cause mortality in a US population: results from the NIH-AARP Diet and Health Study. *Arch Intern Med.* 2007;167(22):2461–2468. 10.1001/archinte.167.22.2461 18071168

[64] Jin Q Black A Kales SN: Metabolomics and Microbiomes as Potential Tools to Evaluate the Effects of the Mediterranean Diet. *Nutrients.* 2019;11(1): pii: E207. 10.3390/nu11010207 30669673 PMC6356665

[65] Zhao L Zhang F Ding X: Gut bacteria selectively promoted by dietary fibers alleviate type 2 diabetes. *Science.* 2018;359(6380):1151–1156. 10.1126/science.aao5774 29590046

[66] Tosti V Bertozzi B Fontana L: Health Benefits of the Mediterranean Diet: Metabolic and Molecular Mechanisms. *J Gerontol A Biol Sci Med Sci.* 2018;73(3):318–326. 10.1093/gerona/glx227 29244059 PMC7190876

[67] Bailey MA Holscher HD: Microbiome-Mediated Effects of the Mediterranean Diet on Inflammation. *Adv Nutr.* 2018;9(3):193–206. 10.1093/advances/nmy013 29767701 PMC5952955

[68] David LA Maurice CF Carmody RN: Diet rapidly and reproducibly alters the human gut microbiome. *Nature.* 2014;505(7484):559–563. 10.1038/nature12820 24336217 PMC3957428

[69] Richards JL Yap YA McLeod KH: Dietary metabolites and the gut microbiota: an alternative approach to control inflammatory and autoimmune diseases. *Clin Transl Immunology.* 2016;5(5):e82. 10.1038/cti.2016.29 27350881 PMC4910123

[70] Clemente JC Ursell LK Parfrey LW: The impact of the gut microbiota on human health: an integrative view. *Cell.* 2012;148(6):1258–1270. 10.1016/j.cell.2012.01.035 22424233 PMC5050011

[71] Tang WH Wang Z Levison BS: Intestinal microbial metabolism of phosphatidylcholine and cardiovascular risk. *N Engl J Med.* 2013;368(17):1575–1584. 10.1056/NEJMoa1109400 23614584 PMC3701945

[72] Schugar RC Shih DM Warrier M: The TMAO-Producing Enzyme Flavin-Containing Monooxygenase 3 Regulates Obesity and the Beiging of White Adipose Tissue. *Cell Rep.* 2017;19(12):2451–2461. 10.1016/j.celrep.2017.05.077 28636934 PMC5672822

[73] Zhu W Gregory JC Org E: Gut Microbial Metabolite TMAO Enhances Platelet Hyperreactivity and Thrombosis Risk. *Cell.* 2016;165(1):111–124. 10.1016/j.cell.2016.02.011 26972052 PMC4862743

[74] Thorburn AN Macia L Mackay CR: Diet, metabolites, and "western-lifestyle" inflammatory diseases. *Immunity.* 2014;40(6):833–842. 10.1016/j.immuni.2014.05.014 24950203

[75] Round JL Mazmanian SK: Inducible Foxp ^3+^ regulatory T-cell development by a commensal bacterium of the intestinal microbiota. *Proc Natl Acad Sci U S A.* 2010;107(27):12204–12209. 10.1073/pnas.0909122107 20566854 PMC2901479

[76] Mazmanian SK Liu CH Tzianabos AO: An immunomodulatory molecule of symbiotic bacteria directs maturation of the host immune system. *Cell.* 2005;122(1):107–118. 10.1016/j.cell.2005.05.007 16009137

[77] Griffin NW Ahern PP Cheng J: Prior Dietary Practices and Connections to a Human Gut Microbial Metacommunity Alter Responses to Diet Interventions. *Cell Host Microbe.* 2017;21(1):84–96. 10.1016/j.chom.2016.12.006 28041931 PMC5234936

[78] Sonnenburg ED Smits SA Tikhonov M: Diet-induced extinctions in the gut microbiota compound over generations. *Nature.* 2016;529(7585):212–215. 10.1038/nature16504 26762459 PMC4850918

[79] Dey N Wagner VE Blanton LV: Regulators of gut motility revealed by a gnotobiotic model of diet-microbiome interactions related to travel. *Cell.* 2015;163(1):95–107. 10.1016/j.cell.2015.08.059 26406373 PMC4583712

[80] Delgado-Lista J Perez-Martinez P Garcia-Rios A: CORonary Diet Intervention with Olive oil and cardiovascular PREVention study (the CORDIOPREV study): Rationale, methods, and baseline characteristics: A clinical trial comparing the efficacy of a Mediterranean diet rich in olive oil versus a low-fat diet on cardiovascular disease in coronary patients. *Am Heart J.* 2016;177:42–50. 10.1016/j.ahj.2016.04.011 27297848 PMC4910622

[81] Haro C Montes-Borrego M Rangel-Zúñiga OA: Two Healthy Diets Modulate Gut Microbial Community Improving Insulin Sensitivity in a Human Obese Population. *J Clin Endocrinol Metab.* 2016;101(1):233–242. 10.1210/jc.2015-3351 26505825

[82] Karlsson FH Tremaroli V Nookaew I: Gut metagenome in European women with normal, impaired and diabetic glucose control. *Nature.* 2013;498(7452):99–103. 10.1038/nature12198 23719380

[83] Qin J Li Y Cai Z: A metagenome-wide association study of gut microbiota in type 2 diabetes. *Nature.* 2012;490(7418):55–60. 10.1038/nature11450 23023125

[84] Carmody RN Gerber GK Luevano JM Jr: Diet dominates host genotype in shaping the murine gut microbiota. *Cell Host Microbe.* 2015;17(1):72–84. 10.1016/j.chom.2014.11.010 25532804 PMC4297240

[85] Yatsunenko T Rey FE Manary MJ: Human gut microbiome viewed across age and geography. *Nature.* 2012;486(7402):222–7. 10.1038/nature11053 22699611 PMC3376388

[86] Flint HJ: The impact of nutrition on the human microbiome. *Nutr Rev.* 2012;70 Suppl 1:S10–S13. 10.1111/j.1753-4887.2012.00499.x 22861801

[87] Turnbaugh PJ Ridaura VK Faith JJ: The effect of diet on the human gut microbiome: a metagenomic analysis in humanized gnotobiotic mice. *Sci Transl Med.* 2009;1(6):6ra14–16ra14. 10.1126/scitranslmed.3000322 20368178 PMC2894525

[88] De Filippo C Cavalieri D Di Paola M: Impact of diet in shaping gut microbiota revealed by a comparative study in children from Europe and rural Africa. *Proc Natl Acad Sci U S A.* 2010;107(33):14691–14696. 10.1073/pnas.1005963107 20679230 PMC2930426

[89] Moschen AR Wieser V Tilg H: Dietary Factors: Major Regulators of the Gut's Microbiota. *Gut Liver.* 2012;6(4):411–6. 10.5009/gnl.2012.6.4.411 23170142 PMC3493718

[90] Ou J Carbonero F Zoetendal EG: Diet, microbiota, and microbial metabolites in colon cancer risk in rural Africans and African Americans. *Am J Clin Nutr.* 2013;98(1):111–120. 10.3945/ajcn.112.056689 23719549 PMC3683814

[91] Suzuki Y Ikeda K Sakuma K: Association between Yogurt Consumption and Intestinal Microbiota in Healthy Young Adults Differs by Host Gender. *Front Microbiol.* 2017;8:847. 10.3389/fmicb.2017.00847 28553274 PMC5425481

[92] Kišidayová S Váradyová Z Pristas P: Effects of high- and low-fiber diets on fecal fermentation and fecal microbial populations of captive chimpanzees. *Am J Primatol.* 2009;71(7):548–557. 10.1002/ajp.20687 19367605

[93] Cani PD Bibiloni R Knauf C: Changes in gut microbiota control metabolic endotoxemia-induced inflammation in high-fat diet-induced obesity and diabetes in mice. *Diabetes.* 2008;57(6):1470–81. 10.2337/db07-1403 18305141

[94] McCord AI Chapman CA Weny G: Fecal microbiomes of non-human primates in Western Uganda reveal species-specific communities largely resistant to habitat perturbation. *Am J Primatol.* 2014;76(4):347–354. 10.1002/ajp.22238 24285224 PMC4097101

[95] Amato KR Martinez-Mota R Righini N: Phylogenetic and ecological factors impact the gut microbiota of two Neotropical primate species. *Oecologia.* 2016;180(3):717–733. 10.1007/s00442-015-3507-z 26597549

[96] Amato KR Yeoman CJ Cerda G: Variable responses of human and non-human primate gut microbiomes to a Western diet. *Microbiome.* 2015;3:53. 10.1186/s40168-015-0120-7 26568112 PMC4645477

[97] Gomez A Rothman JM Petrzelkova K: Temporal variation selects for diet-microbe co-metabolic traits in the gut of *Gorilla* spp. *ISME J.* 2016;10(2):514–26. 10.1038/ismej.2015.146 26315972 PMC4737941

[98] Gomez A Petrzelkova K Yeoman CJ: Gut microbiome composition and metabolomic profiles of wild western lowland gorillas ( *Gorilla gorilla gorilla*) reflect host ecology. *Mol Ecol.* 2015;24(10):2551–2565. 10.1111/mec.13181 25846719

[99] Clayton JB Vangay P Huang H: Captivity humanizes the primate microbiome. *Proc Natl Acad Sci U S A.* 2016;113(37):10376–10381. 10.1073/pnas.1521835113 27573830 PMC5027417

[100] Hale VL Tan CL Niu K: Diet Versus Phylogeny: a Comparison of Gut Microbiota in Captive Colobine Monkey Species. *Microb Ecol.* 2018;75(2):515–527. 10.1007/s00248-017-1041-8 28735426

[101] Jasinska AJ Schmitt CA Service SK: Systems biology of the vervet monkey. *ILAR J.* 2013;54(2):122–143. 10.1093/ilar/ilt049 24174437 PMC3814400

[102] Phillips KA Bales KL Capitanio JP: Why primate models matter. *Am J Primatol.* 2014;76(9):801–827. 10.1002/ajp.22281 24723482 PMC4145602

[103] Ley RE Hamady M Lozupone C: Evolution of mammals and their gut microbes. *Science.* 2008;320(5883):1647–1651. 10.1126/science.1155725 18497261 PMC2649005

[104] Bäckhed F Ley RE Sonnenburg JL: Host-bacterial mutualism in the human intestine. *Science.* 2005;307(5717):1915–1920. 10.1126/science.1104816 15790844

[105] Ley RE Peterson DA Gordon JI: Ecological and evolutionary forces shaping microbial diversity in the human intestine. *Cell.* 2006;124(4):837–848. 10.1016/j.cell.2006.02.017 16497592

[106] Amato KR Yeoman CJ Kent A: Habitat degradation impacts black howler monkey ( *Alouatta pigra*) gastrointestinal microbiomes. *ISME J.* 2013;7(7):1344–53. 10.1038/ismej.2013.16 23486247 PMC3695285

[107] Sánchez-Villegas A Delgado-Rodríguez M Alonso A: Association of the Mediterranean dietary pattern with the incidence of depression: the Seguimiento Universidad de Navarra/University of Navarra follow-up (SUN) cohort. *Arch Gen Psychiatry.* 2009;66(10):1090–1098. 10.1001/archgenpsychiatry.2009.129 19805699

[108] Ley RE: Obesity and the human microbiome. *Curr Opin Gastroenterol.* 2010;26(1):5–11. 10.1097/MOG.0b013e328333d751 19901833

[109] Wu GD Chen J Hoffmann C: Linking long-term dietary patterns with gut microbial enterotypes. *Science.* 2011;334(6052):105–108. 10.1126/science.1208344 21885731 PMC3368382

[110] Mackie RI Aminov RI Hu W: Ecology of uncultivated *Oscillospira* species in the rumen of cattle, sheep, and reindeer as assessed by microscopy and molecular approaches. *Appl Environ Microbiol.* 2003;69(11):6808–6815. 10.1128/aem.69.11.6808-6815.2003 14602644 PMC262257

[111] Haro C Montes-Borrego M Rangel-Zúñiga OA: Two Healthy Diets Modulate Gut Microbial Community Improving Insulin Sensitivity in a Human Obese Population. *J Clin Endocrinol Metab.* 2016;101(1):233–242. 10.1210/jc.2015-3351 26505825

[112] Haro C Garcia-Carpintero S Alcala-Diaz JF: The gut microbial community in metabolic syndrome patients is modified by diet. *J Nutr Biochem.* 2016;27:27–31. 10.1016/j.jnutbio.2015.08.011 26376027

[113] Miquel S Martín R Rossi O: *Faecalibacterium prausnitzii* and human intestinal health. *Curr Opin Microbiol.* 2013;16(3):255–261. 10.1016/j.mib.2013.06.003 23831042

[114] Videla S Vilaseca J Antolín M: Dietary inulin improves distal colitis induced by dextran sodium sulfate in the rat. *Am J Gastroenterol.* 2001;96(5):1486–1493. 11374687 10.1111/j.1572-0241.2001.03802.x

[115] Costabile A Klinder A Fava F: Whole-grain wheat breakfast cereal has a prebiotic effect on the human gut microbiota: a double-blind, placebo-controlled, crossover study. *Br J Nutr.* 2008;99(1):110–120. 10.1017/S0007114507793923 17761020

[116] Ben XM Zhou XY Zhao WH: Supplementation of milk formula with galacto-oligosaccharides improves intestinal micro-flora and fermentation in term infants. *Chin Med J (Engl).* 2004;117(6):927–931. 15198901

[117] Ramirez-Farias C Slezak K Fuller Z: Effect of inulin on the human gut microbiota: stimulation of *Bifidobacterium adolescentis* and *Faecalibacterium prausnitzii*. *Br J Nutr.* 2009;101(4):541–550. 10.1017/S0007114508019880 18590586

[118] Pusceddu MM El Aidy S Crispie F: N-3 Polyunsaturated Fatty Acids (PUFAs) Reverse the Impact of Early-Life Stress on the Gut Microbiota. *PLoS One.* 2015;10(10):e0139721. 10.1371/journal.pone.0139721 26426902 PMC4591340

[119] Robertson RC Seira Oriach C Murphy K: Omega-3 polyunsaturated fatty acids critically regulate behaviour and gut microbiota development in adolescence and adulthood. *Brain Behav Immun.* 2017;59:21–37. 10.1016/j.bbi.2016.07.145 27423492

[120] Shively CA Register TC Appt SE: Consumption of Mediterranean versus Western Diet Leads to Distinct Mammary Gland Microbiome Populations. *Cell Rep.* 2018;25(1):47–56.e43. 10.1016/j.celrep.2018.08.078 30282037 PMC6338220

[121] Caesar R Tremaroli V Kovatcheva-Datchary P: Crosstalk between Gut Microbiota and Dietary Lipids Aggravates WAT Inflammation through TLR Signaling. *Cell Metab.* 2015;22(4):658–668. 10.1016/j.cmet.2015.07.026 26321659 PMC4598654

[122] Lecomte V Kaakoush NO Maloney CA: Changes in gut microbiota in rats fed a high fat diet correlate with obesity-associated metabolic parameters. *PLoS One.* 2015;10(5):e0126931. 10.1371/journal.pone.0126931 25992554 PMC4436290

[123] Shively CA Appt SE Vitolins MZ: Mediterranean versus Western Diet Effects on Caloric Intake, Obesity, Metabolism, and Hepatosteatosis in Nonhuman Primates. *Obesity (Silver Spring).* 2019;27(5):777–784. 10.1002/oby.22436 31012294 PMC7079682

[124] Cani PD: Human gut microbiome: hopes, threats and promises. *Gut.* 2018;67(9):1716–1725. 10.1136/gutjnl-2018-316723 29934437 PMC6109275

[125] Gutiérrez-Díaz I Fernández-Navarro T Sánchez B: Mediterranean diet and faecal microbiota: a transversal study. *Food Funct.* 2016;7(5):2347–2356. 10.1039/c6fo00105j 27137178

[126] Yadav H Jain S Bissi L: Gut microbiome derived metabolites to regulate energy homeostasis: how microbiome talks to host. *Metabolomics.* 2016;6:e150 10.4172/2153-0769.1000e150

