# Supplementary material for: Gut microbiome-Mediterranean diet interactions in improving host health
Source: F1000Res. 2019 May 21;8:699. [Version 1] doi: 10.12688/f1000research.18992.1 (PMC7359750; doi:10.12688/f1000research.18992.1)
Supplement: Peer review history

# Peer review: Reviewer response for version 1

**DOI:** 10.5256/f1000research.20817.r65807

**Published:** 2020-08-21

**Authors:** Raghvendra Kumar Mishra

**Affiliations:** 1 Amity Institute of Biotechnology, Amity University Gwalior, Gwalior, Madhya Pradesh, India

This manuscript describes very timely and important review on how diet and microbiome interactions play a crucial role in maintaining health, especially mental health. This manuscript has been well written, cited all important references properly, and easy to read with nice graphic. I strongly believe that indexing this manuscript on time will provide high readership to the journal.

 I find this topic very interesting with well written paper, thus recommend indexing of this interesting manuscript immediately.

Is the topic of the opinion article discussed accurately in the context of the current literature?

Yes

Are arguments sufficiently supported by evidence from the published literature?

Yes

Are all factual statements correct and adequately supported by citations?

Yes

Are the conclusions drawn balanced and justified on the basis of the presented arguments?

Yes

Reviewer Expertise:

1. Arachidonic acid production and molecular characterization of Mortierella alipna isolates (Filed 02 patents, 01 paper, 01 submitted). 2. Biosynthesis of Nanoparticles from C. rosesus (01 paper published in Frontiers in Microbiology other in pipe line). 3. Development of EST-SSR and ILP data for commercially important plan variety. (03 papers published, one project is going on)

I confirm that I have read this submission and believe that I have an appropriate level of expertise to confirm that it is of an acceptable scientific standard.

# Peer review: Reviewer response for version 1

**DOI:** 10.5256/f1000research.20817.r65835

**Published:** 2020-07-13

**Authors:** Rajeev Kapila

**Affiliations:** 1 Animal Biochemistry Division, ICAR-National Dairy Research Institute, Karnal, Haryana, India

In the current perspective, the authors have lucidly briefed that shifting of dietary-pattern from ‘Western-style’ dietary habits to a ‘Mediterranean-style‘diet effectively target gut microbiome interactions by diversifying and increasing the richness of gut microbiome which can justifiably help in prevention and treatment of various chronic diseases like obesity, diabetes, cancer and aging-related diseases such as Alzheimer’s disease. In a quintessential and step-by-step manner, the authors have well described the usefulness of the Mediterranean-style diet which represents a promising, efficacious, and holistic approach to restore host heath. 

 In this article, the authors have discussed the nutritional characteristics of a typical Mediterranean-style dietary pattern along with their components having an association with various health benefits. Briefly discussed the adverse effects of TMAO released from Western-style diet over beneficial effects of SCFA released during dietary fermentation of complex carbohydrates present in Mediterranean-diet. Thus authors have also suggested some underlying mechanisms by which the purported health benefits of MD can be exerted. The authors have justified the use of non-human primates as an ideal model to dissect diet-microbiome interactions in human health and disease after drawing evidence from the fact that the diet-microbiome network is universally consistent in human and animal studies. In a nutshell, the manuscript is nicely written with sufficient facts and figures and the following comments are suggested for each section of the manuscript wherever they may apply

**Comments:**

1. It could be better if a tabular description of Western-style’ dietary habits and their components would also be made highlighting the difference in components.
2. Release of metabolites such as SCFA is now well recognised as epigenetic modifiers which could be an alternative underlying mechanism of beneficial effects of Mediterranean-style dietary pattern due to the abundance of prebiotic and polyphenolic components which may also be highlighted in proposed biochemical pathways.
3. Writing “Mediterranean dietary pattern” as rich in minimally processed or unprocessed food, unlike a Western-style dietary pattern appears faulty because cooking and frying used in these dietary patterns are also a form of food processing hence it is suggested to use different terminology to differentiate between processed / un-processed/ cooked foods.  
4. In the introduction section sentence “mono- and poly-unsaturated fatty acids such as fish and olive oil” could be written as mono- and poly-unsaturated fatty acids consumed through fish and olive oil.
5. Under the heading “Mediterranean diet: the intangible cultural and nutritional dietary pattern” the sentence “A Mediterranean-style diet typifies a nutritionally balanced diet, characterized by intake in high amounts and frequency of important sources of fibers” may be modified as “A Mediterranean-style diet typifies a nutritionally balanced diet, characterized by high and frequent intake of important sources of fibres”.
6. It would be better if some references are cited with “Table 1”, if available.
7. In Table 2, Figure 1 and 2, use different yet consistent colours for ↓↑ respectively.
8. Under the heading “Microbiome-mediated pro-health effects of MD: purported and speculated mechanisms” the sentence “Thus, the array of microbial metabolites in the host gut from dietary fermentation…... is too long hence better to modify for more clarity.
9. Similarly under the heading “Effect of MD on NHP microbiome: similar findings as seen in human and rodent studies” the sentence  “Altogether, these findings from our cohort of the MD-fed NHPs demonstrate that the positive modulation of the gut microbiome....... is very lengthy may be re-written for better understanding

Is the topic of the opinion article discussed accurately in the context of the current literature?

Yes

Are arguments sufficiently supported by evidence from the published literature?

Yes

Are all factual statements correct and adequately supported by citations?

Yes

Are the conclusions drawn balanced and justified on the basis of the presented arguments?

Yes

Reviewer Expertise:

Health validation of nutraceuticals (Probiotics and  Bioactive peptides) and their  mode of actions

I confirm that I have read this submission and believe that I have an appropriate level of expertise to confirm that it is of an acceptable scientific standard.

# Peer review: Reviewer response for version 1

**DOI:** 10.5256/f1000research.20817.r52604

**Published:** 2019-10-15

**Authors:** Prakash Halami

**Affiliations:** 1 Department of Microbiology and Fermentation Technology, CSIR-Central Food Technological Research Institute, Mysore, Karnataka, India

In this article the authors have summarized the importance of consuming a Mediterranean-style diet, which is associated with prevention of metabolic and cardiovascular diseases. It is know that a Mediterranean-style diet (MD) contributes to the high proportion of fibre, mono- and poly-unsaturated fatty acids, antioxidants and polyphenols. The authors have also stated the beneficial modulation of the gut microbiome is associated with MD as evident from in-vivo **experimental studies.

 The consumption of MD has provided many health benefits which is associated with rich microbial diversity, composition and its function. In this review article, the author have discussed, with evidence, the MD in rodent & human subjects, as well as the importance of gut bacteria and microbial metabolites. The article has been well-written with reference to bile, gut microbiota & chronic diseases, where in authors have underlined the benefits of MD. They have also summarised western style of dietary habits associated with chronic diseases.

 Further, the authors have provided information on MD, its intangible culture and nutritional dietary pattern along with the ingredients generally used. In subsequent sections, MD and its positive health effects and possible mechanisms is provided in a tabulated form. The authors have presented a diagrammatic overview of the consumption of MD and its effect on gut microbiota, especially on microbiome diversity by minimizing the occurrence of firmicutes and proteobacteria. In subsequent sections the authors have mentioned the microbial mediated beneficial effects of MD and it possible mechanisms. The authors have mentioned the use of Non-human Primates (NHP) for studying diet microbial interaction with reference to human health and diseases. In subsequent sections, the authors have mentioned the effects of MD on the microbial diversity of NHP and its correlation with human subjects, distinct microflora associated with human subjects e.g. *Ruminococcus* is also described.

 In the 'Perspectives on the future' section the authors have highlighted the importance of plant based foods and its importance to host metabolic rate etc. The microbiome composition and metabolite production that are required to prevent various lifestyle disorders have been suggested. Authors have also provided information on how probiotics can influence the gut microbiome of the personalized hot. Accordingly, authors have prepared the article well and following comments are suggested:    

 Comments:

1. The use of the acronym MD for "Mediterranean-style diet" should be consistent throughout the MS.
2. In table 2, please change "SCFA" in possible mechanism to "SCFA production".
3. The many repeated MD ingredients mentioned in table 2 could be better represented by combining a few of the rows.
4. In figure 1, it is suggested that the font colour for the increased parameters could be green and the those for decreased one could be red.
5. Authors could include a section for the role of prebiotic compounds that are found in MD.
6. A concern when comparing NHP microbiome with that of the human microbiome is that is all the interaction (enzymatic or hormonal) similar to that of NHP? As any difference in these factors in the two species can result in misleading conclusions.

Is the topic of the opinion article discussed accurately in the context of the current literature?

Yes

Are arguments sufficiently supported by evidence from the published literature?

Yes

Are all factual statements correct and adequately supported by citations?

Yes

Are the conclusions drawn balanced and justified on the basis of the presented arguments?

Partly

Reviewer Expertise:

Probiotics, gut microbiota, antibiotic resistance in lactic acid bacteria & antimicrobial peptides

I confirm that I have read this submission and believe that I have an appropriate level of expertise to confirm that it is of an acceptable scientific standard.
